# Individual and community level predictors of utilization of deworming medications among pregnant women in Ethiopia: A multilevel analysis

**DOI:** 10.1371/journal.pntd.0010731

**Published:** 2022-09-15

**Authors:** Fantu Mamo Aragaw, Daniel Gashaneh Belay, Mastewal Endalew, Melaku Hunie Asratie, Moges Gashaw, Nuhamin Tesfa Tsega

**Affiliations:** 1 Department of Epidemiology and Biostatistics, Institute of Public Health, College of Medicine and Health Sciences, University of Gondar, Gondar, Ethiopia; 2 Department of Human Anatomy, College of Medicine and Health Sciences, University of Gondar, Gondar, Ethiopia; 3 Department of Environmental and Occupational Health and Safety, Institute of Public Health, College of Medicine and Health Sciences, University of Gondar, Gondar,Ethiopia; 4 Department of Women’s and Family Health, School of Midwifery, College of Medicine and Health Sciences, University of Gondar, Gondar, Ethiopia; 5 Department of physiotherapy, College of Medicine and Health Sciences, University of Gondar, Gondar, Ethiopia; Ben-Gurion University of the Negev, ISRAEL

## Abstract

**Background:**

Deworming is one strategy for reducing the burden of anaemia in pregnant women caused by intestinal parasites and it is one of the components of prenatal treatment offered to pregnant women in Ethiopia during antenatal care visits. However, there is limited evidence on the levels of deworming utilization and its determinants in Ethiopia. Hence, this study was aimed to assess the levels of deworming utilization and its individual and community level determinants among pregnant women in Ethiopia.

**Method:**

This study used a total weighted sample of 7590 reproductive-aged women who gave birth in the five years preceding the survey from the 2016 EDHS data. The data were cleaned and weighted using STATA version 16. Results were presented with tables and texts. Individual and community level determinants for deworming use among Ethiopian pregnant women were identified using a multilevel binary logistic regression model. In the multivariable multilevel analysis, those variables with *p*-value < 0.05 were considered to be significantly associated with utilization of deworming medication and reported with adjusted odd ratio with 95% confidence level.

**Results:**

The overall utilization of deworming among pregnant women was 5.69% (95% CI: 5.24%, 6.33) in Ethiopia. Having occupation [AOR = 1.59; 95% CI; 1.27, 1.99], wanted pregnancy [AOR = 1.51; 95% CI; [1.16, 1.95], having ANC visit [AOR = 2.72; 95% CI; 2.03,3.64], media exposure [AOR = 1.67; 95%CI; 1.30,2.15], and high community level poverty [AOR = 0.59; 95% CI; 0.40,0.87] were significantly associated with utilization of deworming among pregnant women’s.

**Conclusion:**

According to the findings of this study, out of twenty pregnant women, only one pregnant woman utilizes deworming medication in Ethiopia. Pregnant woman having an occupation, being exposed for media, having wanted pregnancy, having ANC visits and live with low level community poverty were more likely to use deworming medication. Therefore, intervention efforts to enhance utilization of deworming in Ethiopia requires working on enabling factors like media exposure, ANC visit and pregnancy desirability. In addition, Furthermore, increasing the community’s economic capacity could support in increasing deworming medication uptake.

## Author summary

Neglected intestinal parasitic infections, such as soil-transmitted helminthes, have been identified as a major public health issue in the world’s poorest communities. Women of reproductive age, especially pregnant women, are at a particular risk from soil transmitted helminthiasis which results in morbidities such as anemia. Deworming is one strategy for reducing the burden of intestinal parasites in pregnant women. Little is known about levels of deworming utilization and its determinants among pregnant women in Ethiopia. We used national survey data from approximately 7590 pregnant women to assess deworming utilization and its individual and community-level determinants in Ethiopia. We found that the level of utilization of deworming was very low, there is a higher uptake of deworming medication among womens who have an occupation, media exposure, desired pregnancy, ante natal care visit and pregnant women with low level community poverty. Our findings have important implications for scaling up the utilization of anthelminthic treatment for pregnant women.

## Introduction

Intestinal parasite infections (Helminthiasis) are a serious public health concern [1]. Pregnant women are more vulnerable to parasitic infections as a result of their natural immune response to pregnancy [2]. In Ethiopia, a systematic review and meta-analysis study reported a pooled prevalence of intestinal parasites among pregnant women of 29% [3]. Pregnant women with intestinal parasite infections are more likely to have maternal problems and poor perinatal outcomes, such as anemia, low birth weight, and perinatal mortality [4,5].

Intestinal parasitic infections particularly helminthes have been linked with anemia in pregnant women [1,6,7]. Anemia in pregnancy is a significant public health issue around the world [8]. Due to the intensity of anemia in pregnancy, they become more susceptible if they acquire additional infestation of helminthes [2,9–15]. The anemia caused by hookworm and Trichuris Trichiura infections accounts for the majority of this burden [9,16]. Anemia has a number of adverse effects on pregnant women including increased susceptibility to infection, stillbirth/miscarriage [17,18], and poor feto-maternal outcomes such as preterm birth, low birth weight, and infant or child mortality [18].

Soil transmitted helminthes (STH) is a prevalent infection among pregnant women in areas with limited access to sanitation and in communities with low socioeconomic status [6,19,20]. STH becomes more severe and can ultimately results in death in three high-risk groups: pregnant women, preschool children, and school-aged children [21]. Pregnant women have a higher risk than non-pregnant women among women of reproductive age [21].

In areas where Trichuris trichiura and hookworm prevalence is 20% or higher and anemia prevalence among pregnant women is 40% or higher, the World Health Organization (WHO) recommends deworming for pregnant women after the first trimester, using a single dose of mebendazole or albendazole [22–24]. One of the most significant prenatal measures for avoiding anemia in pregnancy is to use deworming medications [25]. Deworming during pregnancy improves birth weight [19,26,27], improves hemoglobin levels and nutritional status [25,28–30], and reduces morbidity and mortality [31].

WHO has set a target for pregnant women to achieve the coverage of deworming at 75% by 2030 [22,32]. Available evidence shows that majority of pregnant women do not receive deworming medication [33]. In a study by Bangret et al in 49 STH endemic countries, the average utilization of deworming was estimated to be 23% [33]. Another study in Cameroon shows only 29.8% of pregnant women receive deworming treatment during pregnancy [34].

Although it is widely believed that deworming can improve anemic status in pregnant women [35,36],still the coverage of deworming utilization among pregnant women in Ethiopia is low. A study done in Ethiopia reported that almost three fourth of pregnant womens do not receive deworming medication [37]. Still, there is lack of evidence on the levels of deworming utilization and its determinants in Ethiopia. Therefore, this study was aimed to assess the levels of deworming utilization and its individual and community level determinants among pregnant women’s in Ethiopia.

## Methods

### Ethics statement

All methods were carried out in accordance with relevant guidelines of the Demographic and Health Surveys (DHS) program. Informed consent was waived from the International Review Board of Demographic and Health Surveys (DHS) program data archivists, after the consent paper was submitted to DHS Program, a letter of permission to download the dataset for this study. The dataset was not shared or passed on to other bodies and has maintained its confidentiality.

### Study design, setting, and period

A cross-sectional survey study design was undertaken based on 2016 EDHS. Ethiopia is an East African country with a population of 120,499,230 people, making it the second most populous country in Africa [38]. Administratively, Ethiopia is decentralized at the federal level, subdivided with nine regions and two city administrations and regions are divided into zones, and zones, into administrative units called Woreda. Each Woreda is further subdivided into Kebeles, which are the smallest administrative entities. Kebele is also subdivided into census enumeration areas (EAs), which are convenient for the implementation of census. The detail of the study design and setting are detailed elsewhere in Central Statistical Agency (CSA) Ethiopia [39].

### Source and study population

All reproductive age women [15–49] in Ethiopia were the source population. The study population consisted of women who had given birth within the last five years.

### Sample size and sampling procedure

This study was based on the Ethiopian demographic and health survey (EDHS) 2016 data which was a nationally representative sample conducted from January 18, 2016, to June 27, 2016. To select enumeration areas for EDHS 2016, a total of 84,915 Enumeration areas (EAs) from an Ethiopian Population and Housing Census (PHC) conducted in 2007 were used as a sampling frame. The survey used a stratified two-stage cluster sampling procedure as a sampling method. In the first stage a total of 645 EAs (443 in rural areas) and in the second stage an average of 28 households per each cluster were chosen. Any further information about the data/survey exists in the 2016 EDHS report [40].

For this study, we have used the individual record data set and the study population was women (aged 15 to 49 years) who gave birth five years preceding the survey. Weighted values were used to maintain the representativeness of the sample data and generated using individual’s record (IR) EDHS datasets. Finally, this study included a total weighted sample of 7,590 participants was included for this study.

### Outcome variable

The outcome variable of this study was utilization of deworming medication during pregnancy which has a binary response. The DHS asked pregnant women who took deworming medication with a birth in the last five years [41]. A woman said taking drugs for intestinal parasites during pregnancy coded as “1”, and women said not taking drugs for intestinal parasites during pregnancy coded it as “0”.

### Independent variables

Independent variables at the individual and community levels were considered. Individual socio-demographic variables such as maternal age, occupational status of women, marital status, maternal education, household size, sex of household head, household wealth and distance from the health facility were included in the individual-level factors. Characteristics of pregnancy such as parity and pregnancy desirability were also considered. Finally, behavioural characteristics like media exposure were included. Media exposure status is created from the frequency of reading a newspaper or magazine, watching TV, and listening to the radio. If a woman has at least one yes, she has considered having media exposure.

Residence, region, community-level media exposure, community level women education and community level poverty were all considered as community-level determinants. The level of poverty in the community was determined by the proportion of women in the poorest and poorest quintiles obtained from the wealth Index results and classified as low (communities in which < 50% women had poor and poorest wealth quintiles) and high (communities in which ≥50% women had poorest and poorer wealth quintiles) poverty communities. Aggregate values measured by the proportion of women with a minimum of primary level of education derived from data on respondents’ level of education. Then, it was categorized using national median value to values: low (communities with <50% of women have at least primary education) and high (communities with ≥ 50% of women have at least primary education) community level of women education. A community-level media exposure measured by the proportion of women who had exposed to at least one media; television, radio, or newspaper and classified based on national median value as low (communities with <50% of women exposed) and high (communities with ≥50% of women exposed) [42,43].

### Data management and analysis

This study was conducted using data from the three EDHSs received from the official DHS measure website www.measuredhs.com. We extracted the outcome and independent variables from the collection of Child data (KR) data [39]. Based on the Guide to DHS Statistics in STATA version 16, data was cleaned and recoded. Before conducting any statistical analysis, we weighted for sample probabilities and non-response using the weighting factor provided in the EDHS data, as per the survey report’s suggestion, to restore the survey’s representativeness and obtain valid statistical estimates.

### Model building

Four models were fitted in the multi-level study. The first was a null model that only contained outcome variables and was used to examine the variability of deworming utilization in the community. Individual-level factors and community-level variables are included in the second (model 2) and third (model 3) hierarchical models, respectively. Both community and individual level variables with deworming usage were fitted simultaneously in the fourth model (Model 4).

The variance inflation factor (VIF) was used to detect multicollinearity, and all variables had VIF values less than 10 and the mean VIF value of the final model was 1.63.

### Parameter estimation method

Fixed effects (a measure of association) were used to assess the relationship between likelihood of utilization of deworming medication among pregnant women and explanatory variables at both individual and community levels. Variables with a significant association in Adjusted Odds Ratio (AOR) ratios were declared with a p-value of 0.05 and 95 percent confidence intervals in the fixed effect measure of association. The random effect which used to measure the variation was estimated using the median odds ratio (MOR), Intra Class Correlation Coefficient (ICC), and Proportional Change in Variance (PCV). MOR is defined as the central value of the odds ratio between regions highest risk and the lowest risk when randomly picking out two clusters. The PCV reveals the variation in utilization of deworming among pregnant women explained by factors. The ICC displays the differences in deworming usage among pregnant women between clusters [42,44].

## Results

### Sociodemographic characteristics of the study population

A total weighted sample of 7,590 pregnant women was included in this study. Among these pregnant women’s, almost half, 3,827 (50.42%) were between the ages of 25 and 34 years. Majority of the research participants, 6,621 (87.23%) lived in rural areas and about two third, 4,791 (63.12%) of the women had no formal education **[Tables 1 and 2]**.

**Table 1:** Sociodemographic and economic characteristics for utilization of deworming among pregnant women, 2016 EDHS.

Variables	Categories	Utilization of deworming during pregnancy		Total weighted frequency (%)
		Yes (%) n = 432(5.69)	No (%) n = 7,158 (94.31)	
Age of women	15–24	100(5.54)	1,704 (46.96)	1,804 (23.77)
	25–34	235 (6.13)	3,592 (93.87)	3,827 (50.42)
	35–49	98 (49.84)	1,862 (95.03)	1,960 (25.82)
Marital status	Married	398 (5.66)	6,622(94.34)	7,020(92.49)
	Not married	35(6.06)	535(93.94)	570(7.51)
Women education status	No education	229(4.79)	4,562(95.21)	4,791(63.12)
	Primary	1996(92.86)	153(7.14)	2,149(28.32)
	Secondary	385(91.79)	34(8.21)	419(5.53)
	Higher	214(93.49)	14(6.51)	229 (3.02)
Husbands educational status	No education	165(4.86)	3,224(95.14)	3,388(47.67)
	Formal education	243(6.52)	3,478(93.48)	3,719(52.33)
Religion	Orthodox	200(6.96)	2,682(93.04)	2,882(37.97)
	Muslim	135(4.76)	2,689(95.24)	2,824(37.21)
	Protestant	87(5.28)	1,564(94.72)	1,651(21.76)
	Other	9 (4.24)	222(95.76)	232(3.06)
Occupation of women	Not working	175 (4.30)	3,903 (95.70)	4,078(53.73)
	Working	257(7.31)	3,255(92.69)	3,512 (46.27)
Occupation of husband	Not working	19(3.41)	551(96.59)	571(8.03)
	Working	388(5.93)	6,149(94.07)	6,538(91.97)
Household size	1–4	138(5.92)	2,193(94.08)	2,331(30.71)
	5–19	294(5.59)	4,964(94.41)	5,259(69.29)
ANC follow-up	Yes	364(7.65)	4,393(92.35)	4,757(62.67)
	No	2,765(97.59)	68(2.41)	2,833(37.33)
Wanted last birth	Wanted	338(6.07)	5,235(93.93)	5,573(73.43)
	Unwanted	95(4.66)	1,922 (95.34)	2,016(26.57)
Parity	Primi	90(6.21)	1,346(93.79)	1,435(18.90)
	Multi	344(5.57)	5,813(94.43)	6,156 (81.10)
Sex of household head	Male	6,098(94.20)	376 (5.80)	6,474 (85.29)
	Female	57 (5.06)	1,060 (94.94)	1,117 (14.71)
Wealth index	Poor	139(4.20)	3,166 (95.80)	3,305 (43.55)
	Middle	100 (6.23)	1,488(93.68)	1,588 (20.93)
	Rich	192 (7.15)	2,503(92.85)	2,696 (35.52)
Media exposure	No	207(4.17)	4,762 (95.83)	4,969 (65.47)
	Yes	225 (8.59)	2,395 (91.41)	2,620 (34.53)
Distance to health facility	Big problem	220(4.99)	4186(95.01)	4,406(58.06)
	Not big problem	212(6.67)	2,971(93.33)	3,183(41.94)

**Table 2:** Community-level variables for utilization of deworming among pregnant women, EDHS 2016.

Variables	Categories	Utilization of deworming during pregnancy		Total weighted frequency (%)
		Yes (%) n = 432(5.69)	No (%) n = 7,158 (94.31)	
Residence	Urban	75 (7.73)	894 (92.27)	969(12.77)
	Rural	3578(5.40)	6,264 (94.60)	6,621 (87.23)
Community level media exposure	Yes	286(7.19)	3692(92.81)	3,977(52.41)
	No	146 (4.05)	3,466(95.95)	3,612(47.59)
Community level poverty	Low	334(7.19)	4,309(92.81)	4,643(61.18)
	High	98(3.34)	2,848(96.66)	2,946(38.82)
Community Level women education	Low	168(4.47)	3,576(95.53)	3,744(49.33)
	High	265(6.88)	3,581(93.12)	3,845(50.67)
Region	Small peripheral	15(3.47)	426(96.53)	442 (5.81)
	Large central	401 (5.82)	6,498(94.18)	6,899 (90.90)
	Metropolitans	15(6.14)	234(93.86)	249 (3.28)

### Level of deworming utilization

In Ethiopia, the level of deworming utilization was 5.69% (95% CI: 5.24%, 6.33). This deworming utilization varies across different subpopulation. Women who had media exposure, for example, were more likely to use deworming medication (8.59%) than those who had not had media exposure (4.17%). Likewise, women who had an undesirable pregnancy were less likely to use deworming medication (4.66%) than those who had a desired pregnancy (6.07%) **[Table 2]**.

### Model comparison and random effect analysis

As indicated from **Table 3**, the ICC in the null model was 0.21, which means that about 21% of the variations of utilization of deworming medication among pregnant women were attributed to the difference at the cluster level, but the rest 79% were could be atributed to individual level charactherstics. The MOR value was 2.46, in the null model, also indicated that the median odds of utilization of deworming between the lowest and the highest utilization of deworming clusters.

**Table 3:** Results from a random intercept model (a measure of variation) for utilization of deworming among pregnant women at cluster level by multilevel logistic regression analysis, EDHS 2016.

**Random effect**					
	**VA**	0.91	0.71	0.74	0.65
	**ICC**	0.21	0.17	0.18	0.16
	**MOR**	2.46	1.82	2.01	1.65
	**PCV**	----	0.23	0.18	0.29
**Model comparission**					
	**Deviance**	3184	3053	3157	3042
	**Mean VIF**	__	1.41	1.60	1.63

ICC = Inter cluster corrolation cofficent, MOR = Median odds ratio, PCV = proportional change in variance.

Furthermore, the PCV value in the final model (29%) indicates the variation in the utilization of deworming among pregnant study households was explained by both the individual and community level factors. Model comparison/fitness was done using deviance test, then the fourth model has the lowest deviance (3042) and was taken as the best fitted model.

### Predictors of deworming utilization

In the final model analysis variables such as, having occupation, ANC visit, wanted last birth, media exposure status and community level poverty had significant association with deworming utilization among pregnant women.

Based on the final model result, from individual level variables, The odd of deworming utilization in those who have occupation is 1.59 times higher than that of womens who have no occupation [AOR = 1.59;95% CI; 1.27, 1.99]. Pregnant women who have exposure to media have 1.67 times more likely to take deworming medication than those pregnant womenwho have not exposed to media [AOR = 1.67; 95%CI; [1.30,2.15]. The odd of using deworming medication among those who have ANC visit is 2 times higher than that of pregnant womenwho have no ANC visit [AOR = 2.72; 95% CI; [2.03,3.64]. The odd of deworming utilization in those womenswhose last pregnancy was wanted is 1.51 times higher than those women whose pregnancy was not wanted [AOR = 1.51; 95% CI; [1.16, 1.95]. Women who live in high level community poverty are 41% lower odd of deworming utilization than those who live in lower community poverty [AOR = 0.59; 95% CI; [0.40,0.87] [**Table 4**]

**Table 4:** Multilevel multivariable analysis of factors associated with utilization of Deworming Drugs among pregnant women in Ethiopia, EDHS 2016.

Variables	categories		Null model	Model 2		Model 3	Model 4
				AOR [95% CI]		AOR [95% CI]	AOR [95% CI]
Age of women	15–24			1.00			1.00
	25–34			1.03 [0.76, 1.40]			1.03 [0.76,1.41]
	35–49			0.98[0.67, 1.43]			0.97[0.67, 1.42]
Marital status	Not married			1.00			1.00
	Married			0.76[0.49, 1.18]			0.77[0.50,1.18]
Women education status	No education			1.00			1.00
	Primary			1.21 [0.94, 1.57]			1.21 [0.93, 1.57]
	Secondary			1.03 [0.65, 1.63]			1.08[0.67, 1.73]
	Higher			0.64[0.33, 1.21]			0.65[0.35, 1.34]
Religion	Orthodox			1.00			1.00
	Muslim			1.04 [0.75, 1.43]			1.06[0.77, 1.47]
	Protestant			1.03[0.73, 1.47]			0.97[0.68, 1.38]
	Other			1.04[0.48, 2.24]			1.04[0.49, 2.23]
Occupation of women	Not working			1.00			1.00
	Working			1.60[1.28, 2.00]***			1.59[1.27, 1.99]***
Household size	1–4			1.00			1.00
	5–19			1.11 [0.85, 1.45]			1.12[0.86, 1.45]
ANC follow-up	No			1.00			1.00
	Yes			2.77[2.07,3.70]***			2.72[2.03,3.64]***
Wanted last birth	Unwanted			1.00			1.00
	Wanted			1.48[1.14, 1.91]**			1.51[1.16, 1.95]**
Parity	Primi			1.00			1.00
	Multi			1.13 [0.81,1.56]			1.12 [0.81,1.56]
Sex of household head	Male			1.00			1.00
	Female			0.76[0.54,1.07]			0.78[0.55,1.11]
Wealth index	Poor			1.00			1.00
	Middle			1.22 [0.91,1.63]			1.08[0.80,1.46]
	Rich			1.01[0.75,1.34]			0.89[0.65,1.21]
Media exposure	No			1.00			1.00
	Yes			1.70 [1.33,2.17]***			1.67[1.30,2.15]***
Distance to health facility	Big problem			1.00			1.00
	Not big problem			1.01 [0.79,1.28]			0.99[0.78,1.27]
**Community level variables**							
Residence		Rural			1.00		1.00
		Urban			0.83[0.51, 1.33]		0.75[0.45,1.24]
Community level media exposure		No			1.00		1.00
		Yes			1.49[1.05, 2.13]*		1.14[0.80, 1.63]
Community level poverty		Low			1.00		1.00
		High			0.60[0.41,0.88]**		0.59[0.40,0.87]**
Community level women education		Low			1.00		1.00
		High			1.24[0.88,1.74]		1.02 [0.72,1.44]
Region		Small peripheral			1.00		1.00
		Large central			1.19[0.66, 2.15]		1.07 [0.58,1.98]
		Metropolitans			1.03[0.44, 2.45]		0.86[0.36,2.07]

* = P-value < 0.05

** = Pvalue < 0.01

*** = Pvalue < 0.001

AOR = adjusted odds ratio; CI = confidence interval.

## Discussion

This study aimed to identify the individual and community level predictors of deworming utilization among pregnant women in Ethiopia. From our result the utilization of deworming among pregnant women was only 5.69% [5.24%, 6.33] which is very low which directly contributes for the high level helminthic infection among pregnant women in Ethiopia. This result is lower than a study conducted in Tanzania (60.1%) [25], Cameroon(29.8%) [34] and 23% in 49 STH endemic countries [33]. The variations across different countries might be related to factors associated with usage of deworming medication such as socioeconomic factors, difference in study settings, and difference in health care service access and utilization [5,34].

In this study, having occupation for women has a substantial association with utilization of deworming medication. This may be due to the fact that, women who are employed are more likely to attend health care services early than unemployed [45], which is a significant factor for the uptake of deworming drugs.

Also, pregnant women who had ANC had a higher likelihood of taking deworming medication than pregnant women who did not have ANC. This finding is in line with a study done in Cameroon [34]. This may be due to the fact that ANC visit creates a good opportunity to receive deworming medication and more information about benefits of deworming and positive pregnancy outcomes are gained through counseling during repeated ANC visit [24], and WHO recommended to routinely provide deworming medication during ANC visit [22,24], hence a women with ANC visit can easily access the medication and utilize it.

In our study, having wanted pregnancy is also has a higher odds of utilization of deworming medication as compared to women who have unwanted pregnancy. This could be attributed to the fact that, planned pregnancy is associated with early initiation of antenatal services [46],and health-seeking behavior [46], which are important factors for the uptake of the deworming drugs. Also, several studies supported the effect of wanted pregnancy on health care utilization especially for antenatal care services [47–49].

Also, we found higher odds of utilization of deworming medication among pregnant women who were exposed to media, compared to pregnant women who had no media exposure. This finding is consistent with a study done in India [50], and in 26 sub-Saharan countries [5]. Several studies explained the health benefits of media exposure on maternal health care utilization, and birth preparedness [51–53]. This may be due to mothers who are exposed to media have better awareness about the health benefits of medication.

Moreover the study showed lower odds of utilization of deworming medication from higher poverty status in the community. This finding is comparable with a prior study in Ghana [8]. Having a higher community level of poverty is an impediment to easily accessing or affording transportation costs to health services and purchasing medications. Furthermore, higher uptake of ANC service were observed among pregnant women in lower community level poverty as compared to pregnant women in higher community level poverty [54–56].

The strength of this study comes from the use of advanced statistical models that consider individual/household and community level predictors also increase the quality of the paper. However, the study has the following limitations. First, variables not found in the dataset such as drug supply and other variables like perception and attitude were not covered. Secondly, the cross-sectional nature of the data does not allow for a cause-effect relationship. Finally, since the data were self-reported, the finding might be affected by recall bias.

## Conclusion

This study concludes that among twenty pregnant women, only one pregnant woman utilized deworming medication. Pregnant woman having an occupation, being exposed for media, having wanted pregnancy, having ANC visit and pregnant women with low level community poverty were more likely to utilize deworming medication.

Therefore, intervention efforts to enhance utilization of deworming in Ethiopia requires working on enabling factors like media exposure, ANC visit and pregnancy desirability. In addition, improving the economic capacity of the community could help in enhancing uptake of deworming medication.

## References

[1] Crompton DWTJP. The public health importance of hookworm disease. 2000;121(S1):S39–S50. 10.1017/s0031182000006454 11386690

[2] Muhangi L, Woodburn P, Omara M, Omoding N, Kizito D, Mpairwe H, et al. Associations between mild-to-moderate anaemia in pregnancy and helminth, malaria and HIV infection in Entebbe, Uganda. 2007;101(9):899–907. 10.1016/j.trstmh.2007.03.017 PMC1950430 17555783

[3] Chelkeba L, Melaku T, Lemma D, Mekonnen ZJI. Burden of intestinal parasitic infections among pregnant women in Ethiopia: a systematic review and meta-analysis. 2021:1–15. 10.1007/s15010-021-01635-4 34110569

[4] Ahenkorah B, Nsiah K, Baffoe P, Ofosu W, Gyasi C, Owiredu EW. Parasitic infections among pregnant women at first antenatal care visit in northern Ghana: A study of prevalence and associated factors. PloS one. 2020;15(7):e0236514. doi: 10.1371/journal.pone.0236514 32706826 PMC7380595

[5] Zegeye B, Keetile M, Ahinkorah BO, Ameyaw EK, Seidu A-A, Yaya SJTM, et al. Utilization of deworming medication and its associated factors among pregnant married women in 26 sub-Saharan African countries: a multi-country analysis. 2021;49(1):1–15. 10.1186/s41182-021-00343-x PMC8247116 34193313

[6] Brooker S, Hotez PJ, Bundy. Hookworm-related anaemia among pregnant women: a systematic review. 2008;2(9):e291. 10.1371/journal.pntd.0000291 PMC2553481 18820740

[7] Steketee RWJTJon. Pregnancy, nutrition and parasitic diseases. 2003;133(5):1661S–7S. 10.1093/jn/133.5.1661S 12730482

[8] Lumor O, Dzabeng F, Adanu RMJAjorh. Factors influencing the use of anemia preventing measures among antenatal clinic attendees in the Kintampo North Municipality, Ghana. 2019;23(2):35–43. 10.29063/ajrh2019/v23i2.4 31433592

[9] Gyorkos TW, Gilbert NL, Larocque R, Casapía MJTM, Health I. Trichuris and hookworm infections associated with anaemia during pregnancy. 2011;16(4):531–7. 10.1111/j.1365-3156.2011.02727.x 21281406

[10] Fuseinisup G, Edohsup D, Kalifasup BG, Hamidsup A-W, Knight DJJopH, epidemiology. Parasitic infections and anaemia during pregnancy in the Kassena-Nankana district of Northern Ghana. 2010;2(3):48–52.

[11] Larocque R, Casapia M, Gotuzzo E, Gyorkos TWJTAjotm, hygiene. Relationship between intensity of soil-transmitted helminth infections and anemia during pregnancy. 2005;73(4):783–9. 16222026

[12] Ndyomugyenyi R, Kabatereine N, Olsen A, Magnussen PJTotRSoTM, Hygiene. Malaria and hookworm infections in relation to haemoglobin and serum ferritin levels in pregnancy in Masindi district, western Uganda. 2008;102(2):130–6. 10.1016/j.trstmh.2007.09.015 17996912

[13] Nurdiati DS, Sumarni S, Hakimi M, Winkvist AJSAjotm, health p. Impact of intestinal helminth infection on anemia and iron status during pregnancy: a community based study in Indonesia. 2001;32(1):14–22. 11485075

[14] Yatich NJ, Jolly PE, Funkhouser E, Agbenyega T, Rayner JC, Ehiri JE, et al. The effect of malaria and intestinal helminth coinfection on birth outcomes in Kumasi, Ghana. 2010;82(1):28. 10.4269/ajtmh.2010.09-0165 PMC2803505 20064991

[15] Zegeye B, Theresa W, Keetile M, Ahinkorah BO, Ameyaw EK, Seidu A-A, Yaya S, Nicolas L %J Tropical Medicine, et al. Utilization of deworming medicationBlood drain: soil-transmitted helminths and its associated factors among pregnant married women in 26 sub-Saharan African countries: a multi-country analysisanemia in pregnant women. 20212014;498(17):1–15e2912.

[16] Foy H, Nelson GSJEP. Helminths in the etiology of anemia in the tropics, with special reference to hookworms and schistosomes. 1963;14(2):240–62. 10.1016/0014-4894(63)90029-8 14072770

[17] Brabin BJ, Hakimi M, Pelletier DJTJon. An analysis of anemia and pregnancy-related maternal mortality. 2001;131(2S-2):604S–14S; discussion 14S. 10.1093/jn/131.2.604S 11160593

[18] Mawani M, Ali SA, Bano G, Ali SAJRS, Research. SDC. Iron deficiency anemia among women of reproductive age, an important public health problem: situation analysis. 2016;5(3):1.

[19] Walia B, Kmush BL, Lane SD, Endy T, Montresor A, Larsen DAJPNTD. Routine deworming during antenatal care decreases risk of neonatal mortality and low birthweight: a retrospective cohort of survey data. 2021;15(4):e0009282. 10.1371/journal.pntd.0009282 PMC8084140 33914732

[20] Organization WH. Preventive chemotherapy in human helminthiasis. Coordinated use of anthelminthic drugs in control interventions: a manual for health professionals and programme managers: World Health Organization; 2006.

[21] Lo NC, Gupta R, Addiss DG, Bendavid E, Heft-Neal S, Mikhailov A, et al. Comparison of World Health Organization and Demographic and Health Surveys data to estimate sub-national deworming coverage in pre-school aged children. 2020;14(8):e0008551. 10.1371/journal.pntd.0008551 PMC7462292 32804925

[22] Organization WH. Soil-transmitted helminth infections (2020). 2021.

[23] guidelines WHOJDoWn. E-library of Evidence for Nutrition Actions (eLENA). 2013;55:4–6.

[24] Organization WH. WHO recommendations on antenatal care for a positive pregnancy experience: World Health Organization; 2016. 28079998

[25] Yinglan L, Bankanie V, Moshi FV. Factors Associated with Uptake of De-Worming Drugs During Pregnancy Among Women of Reproductive Age in Tanzania; An Analysis of Data from the 2015–16 Tanzania HIV and Malaria Indicators Survey. 2020.

[26] Lau R, Chris RB, Phuong MS, Khatib A, Kopalakrishnan S, Bhasker S, et al. Treatment of soil-transmitted helminth infections in pregnancy: a systematic review and meta-analysis of maternal outcomes. 2020;27(2):taz079. 10.1093/jtm/taz079 31641774

[27] De Silva N, Sirisena J, Gunasekera D, Ismail M, De Silva HJTL. Effect of mebendazole therapy during pregnancy on birth outcome. 1999;353(9159):1145–9. 10.1016/s0140-6736(98)06308-9 10209979

[28] Larocque R, Gyorkos TWJCjoph. Should deworming be included in antenatal packages in hookworm-endemic areas of developing countries? 2006;97(3):222–4. 10.1007/BF03405590 PMC6976180 16827412

[29] Christian P, Khatry SK, West KPJTL Jr. Antenatal anthelmintic treatment, birthweight, and infant survival in rural Nepal. 2004;364(9438):981–3. 10.1016/S0140-6736(04)17023-2 15364190

[30] Atukorala T, De Silva L, Dechering W, Dassenaeike T, Perera RSJTAjocn. Evaluation of effectiveness of iron-folate supplementation and anthelminthic therapy against anemia in pregnancy—a study in the plantation sector of Sri Lanka. 1994;60(2):286–92. 10.1093/ajcn/60.2.286 8030609

[31] Thayer WM, Clermont A, Walker NJBPH. Effects of deworming on child and maternal health: a literature review and meta-analysis. 2017;17(4):113–26. 10.1186/s12889-017-4747-0 PMC5688423 29143641

[32] Organization WH. 2030 targets for soil-transmitted helminthiases control programmes. 2020. 10.1371/journal.pntd.0008505 PMC7446869 32776942

[33] Bangert M, Bancalari P, Mupfasoni D, Mikhailov A, Gabrielli AF, Montresor AJPntd. Provision of deworming intervention to pregnant women by antenatal services in countries endemic for soil-transmitted helminthiasis. 2019;13(5):e0007406. 10.1371/journal.pntd.0007406 PMC6532928 31083673

[34] Zegeye B, Ahinkorah BO, Ameyaw EK, Seidu A-A, Yaya SJBRI. Utilization of Deworming Drugs and Its Individual and Community Level Predictors among Pregnant Married Women in Cameroon: A Multilevel Modeling. 2021;2021. 10.1155/2021/6645336 PMC8137305 34095307

[35] Buseri F, Uko E, Jeremiah Z, Usanga EJTohj. Prevalence and risk factors of anaemia among pregnant women in Nigeria. 2008;2(1).

[36] Damen J, Banwat E, Egah D, Allanana JJAoAM. Parasitic contamination of vegetables in Jos, Nigeria. 2007;6(3):115. 10.4103/1596-3519.55723 18240499

[37] Shiferaw MB, Zegeye AM, Mengistu ADJBrn. Helminth infections and practice of prevention and control measures among pregnant women attending antenatal care at Anbesame health center, Northwest Ethiopia. 2017;10(1):1–5. 10.1186/s13104-017-2609-6 PMC5508608 28701221

[38] Worldometer. Ethiopia Population. 2022.

[39] Central Statistical Agency (CSA) [Ethiopia] and ICF. 2016. Ethiopia Demographic and Health Survey 2016. Addis Ababa, Ethiopia, and Rockville, Maryland, USA: CSA and ICF.

[40] Csa IJE, Calverton. Central Statistical Agency (CSA)[Ethiopia] and ICF. Ethiopia Demographic and Health Survey, Addis Ababa. 2016.

[41] Croft TN, Marshall AM, Allen CK, Arnold F, Assaf S, Balian SJRI. Guide to DHS statistics. 2018;645.

[42] Liyew AM, Teshale AB. Individual and community level factors associated with anemia among lactating mothers in Ethiopia using data from Ethiopian demographic and health survey, 2016; a multilevel analysis. BMC Public Health. 2020;20:1–11. 32448212 10.1186/s12889-020-08934-9 PMC7247135

[43] Teshale AB, Tesema GA. Prevalence and associated factors of delayed first antenatal care booking among reproductive age women in Ethiopia; a multilevel analysis of EDHS 2016 data. PloS one. 2020;15(7):e0235538. doi: 10.1371/journal.pone.0235538 32628700 PMC7337309

[44] Merlo J, Chaix B, Yang M, Lynch J, Råstam L. A brief conceptual tutorial of multilevel analysis in social epidemiology: linking the statistical concept of clustering to the idea of contextual phenomenon. Journal of Epidemiology & Community Health. 2005;59(6):443–9. 15911637 10.1136/jech.2004.023473 PMC1757045

[45] Fagbamigbe AF, Idemudia ESJHcfwi. Wealth and antenatal care utilization in Nigeria: policy implications. 2017;38(1):17–37. 10.1080/07399332.2016.1225743 27537979

[46] Alemu Y, Aragaw AJRh. Early initiations of first antenatal care visit and associated factor among mothers who gave birth in the last six months preceding birth in Bahir Dar Zuria Woreda North West Ethiopia. 2018;15(1):1–8. 10.1186/s12978-018-0646-9 PMC6292069 30541562

[47] Rahman MM, Rahman MM, Tareque MI, Ferdos J, Jesmin SSJPo. Maternal pregnancy intention and professional antenatal care utilization in Bangladesh: a nationwide population-based survey. 2016;11(6):e0157760. 10.1371/journal.pone.0157760 PMC4911017 27309727

[48] Singh A, Singh A, Thapa SJAPJoPH. Adverse consequences of unintended pregnancy for maternal and child health in Nepal. 2015;27(2):NP1481–NP91. 10.1177/1010539513498769 24097931

[49] Hajizadeh M, Nghiem SJIjoph. Does unwanted pregnancy lead to adverse health and healthcare utilization for mother and child? Evidence from low-and middle-income countries. 2020;65(4):457–68. 10.1007/s00038-020-01358-7 PMC7275006 32270238

[50] Ghosh DJTPG. Effect of mothers’ exposure to electronic mass media on knowledge and use of prenatal care services: a comparative analysis of Indian states. 2006;58(3):278–93.

[51] Fatema K, Lariscy JTJS-PH. Mass media exposure and maternal healthcare utilization in South Asia. 2020;11:100614. 10.1016/j.ssmph.2020.100614 PMC7306581 32596437

[52] Asp G, Pettersson KO, Sandberg J, Kabakyenga J, Agardh AJGha. Associations between mass media exposure and birth preparedness among women in southwestern Uganda: a community-based survey. 2014;7(1):22904. 10.3402/gha.v7.22904 PMC3888909 24433945

[53] Zamawe CO, Banda M, Dube ANJBp, childbirth. The impact of a community driven mass media campaign on the utilisation of maternal health care services in rural Malawi. 2016;16(1):1–8. 10.1186/s12884-016-0816-0 PMC4730729 26819242

[54] Arthur E. Wealth and antenatal care use: implications for maternal health care utilisation in Ghana. Health economics review. 2012;2(1):1–8. 22866869 10.1186/2191-1991-2-14 PMC3484029

[55] Fagbamigbe AF, Idemudia ES. Wealth and antenatal care utilization in Nigeria: policy implications. Health care for women international. 2017;38(1):17–37. doi: 10.1080/07399332.2016.1225743 27537979

[56] Shibre G, Zegeye B, Idriss-Wheeler D, Ahinkorah BO, Oladimeji O, Yaya S. Socioeconomic and geographic variations in antenatal care coverage in Angola: further analysis of the 2015 demographic and health survey. BMC public health. 2020;20(1):1–10. 32799833 10.1186/s12889-020-09320-1 PMC7429730

